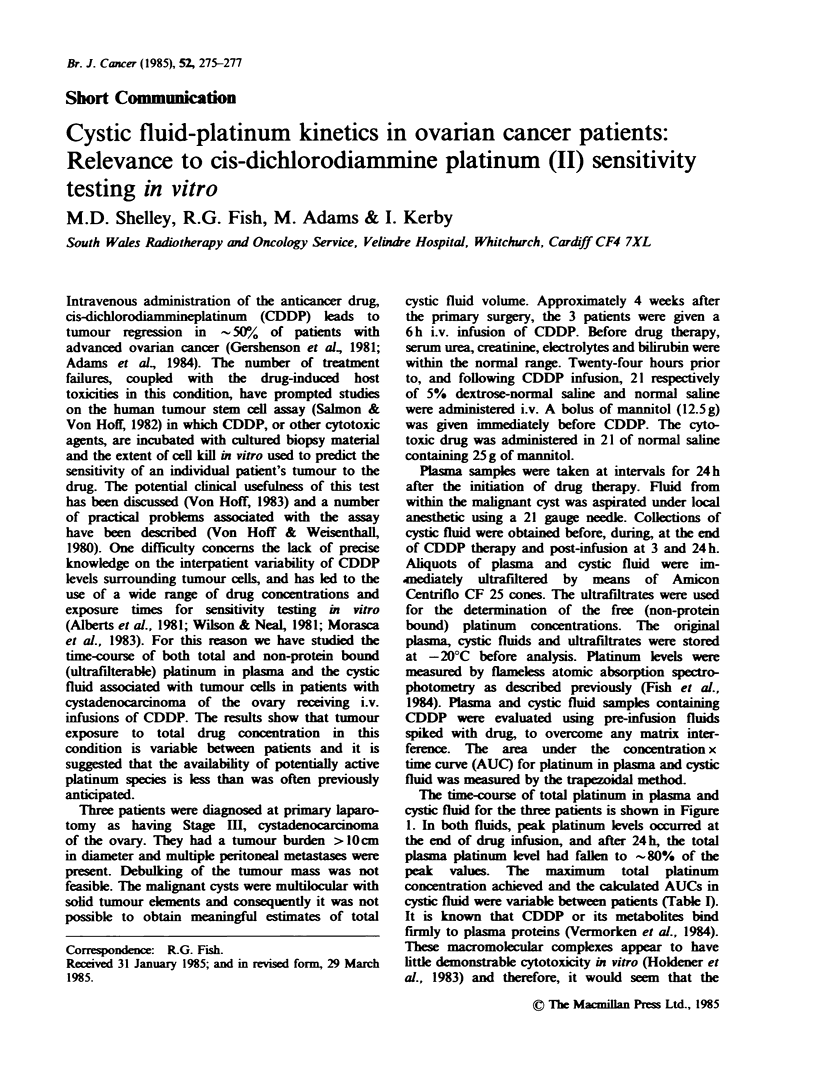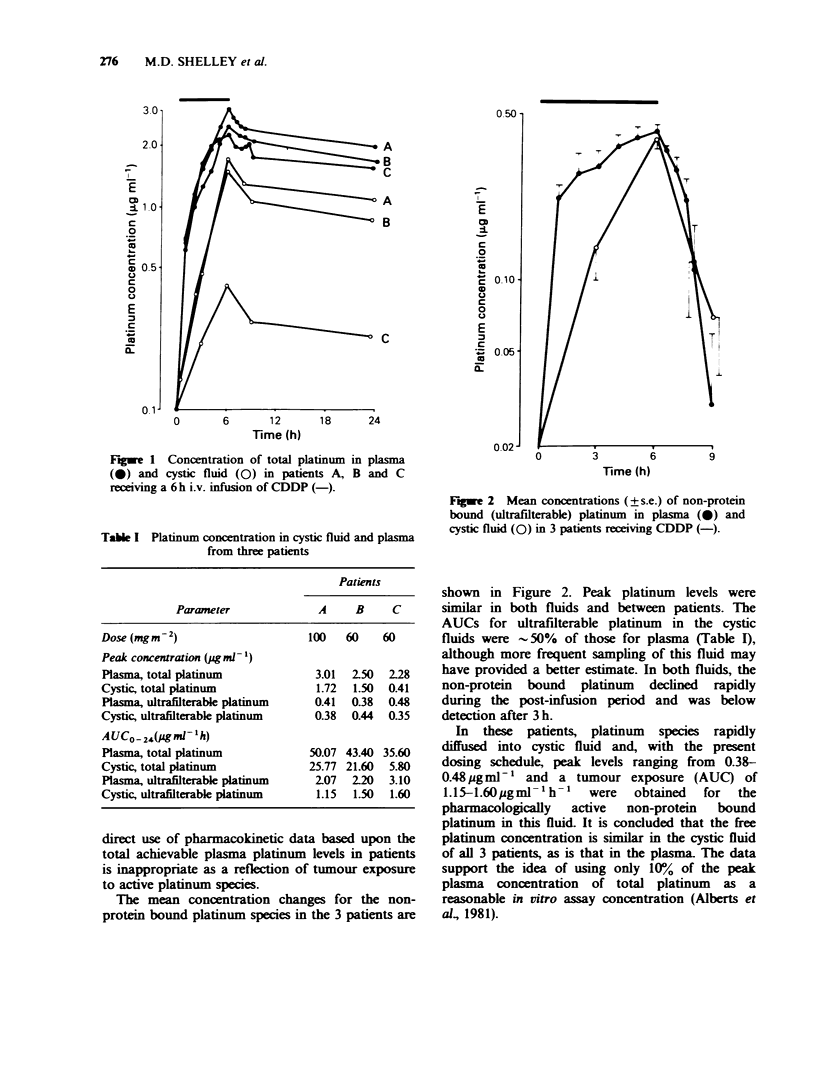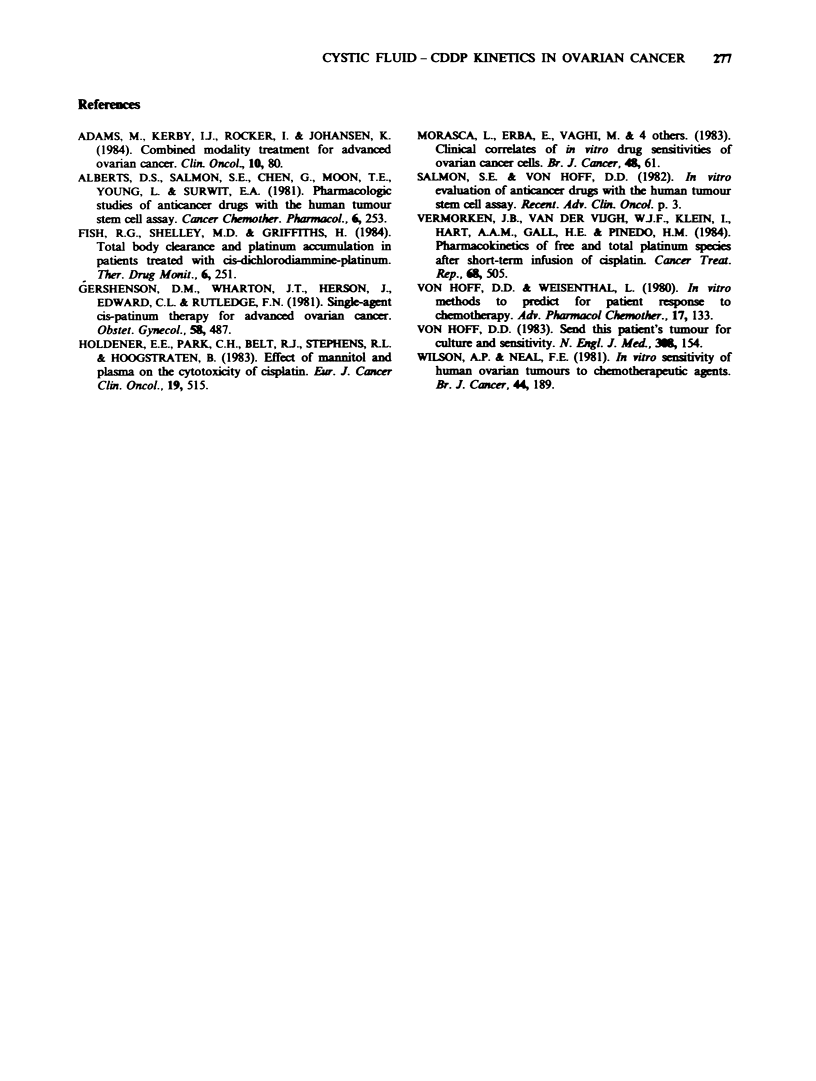# Cystic fluid-platinum kinetics in ovarian cancer patients: relevance to cis-dichlorodiammine platinum (II) sensitivity testing in vitro.

**DOI:** 10.1038/bjc.1985.188

**Published:** 1985-08

**Authors:** M. D. Shelley, R. G. Fish, M. Adams, I. Kerby


*Br. J. Cancer* (1985), **52**, 275–277

**Short Communication**

### Cystic fluid-platinum kinetics in ovarian cancer patients: Relevance to cis-dichlorodiammine platinum (II) sensitivity testing *in vitro*

M.D. Shelley, R.G. Fish, M. Adams & I. Kerby

*South Wales Radiotherapy and Oncology Service, Velindre Hospital, Whitchurch, Cardiff CF4 7XL*

Intravenous administration of the anticancer drug, cis-dichlorodiammineplatinum (CDDP) leads to tumour regression in ~50% of patients with advanced ovarian cancer (Gershenson *et al.*, 1981; Adams *et al.*, 1984). The number of treatment failures, coupled with the drug-induced host toxicities in this condition, have prompted studies on the human tumour stem cell assay (Salmon & Von Hoff, 1982) in which CDDP, or other cytotoxic agents, are incubated with cultured biopsy material and the extent of cell kill *in vitro* used to predict the sensitivity of an individual patient's tumour to the drug. The potential clinical usefulness of this test has been discussed (Von Hoff, 1983) and a number of practical problems associated with the assay have been described (Von Hoff & Weisenthall, 1980). One difficulty concerns the lack of precise knowledge on the interpatient variability of CDDP levels surrounding tumour cells, and has led to the use of a wide range of drug concentrations and exposure times for sensitivity testing *in vitro* (Alberts *et al.*, 1981; Wilson & Neal, 1981; Morasca *et al.*, 1983). For this reason we have studied the time-course of both total and non-protein bound (ultrafilterable) platinum in plasma and the cystic fluid associated with tumour cells in patients with cystadenocarcinoma of the ovary receiving i.v. infusions of CDDP. The results show that tumour exposure to total drug concentration in this condition is variable between patients and it is suggested that the availability of potentially active platinum species is less than was often previously anticipated.

Three patients were diagnosed at primary laparotomy as having Stage III, cystadenocarcinoma of the ovary. They had a tumour burden >10 cm in diameter and multiple peritoneal metastases were present. Debulking of the tumour mass was not feasible. The malignant cysts were multilocular with solid tumour elements and consequently it was not possible to obtain meaningful estimates of total cystic fluid volume. Approximately 4 weeks after the primary surgery, the 3 patients were given a 6 h i.v. infusion of CDDP. Before drug therapy, serum urea, creatinine, electrolytes and bilirubin were within the normal range. Twenty-four hours prior to, and following CDDP infusion, 2 l respectively of 5% dextrose-normal saline and normal saline were administered i.v. A bolus of mannitol (12.5 g) was given immediately before CDDP. The cytotoxic drug was administered in 2 l of normal saline containing 25 g of mannitol.

Plasma samples were taken at intervals for 24 h after the initiation of drug therapy. Fluid from within the malignant cyst was aspirated under local anesthetic using a 21 gauge needle. Collections of cystic fluid were obtained before, during, at the end of CDDP therapy and post-infusion at 3 and 24 h. Aliquots of plasma and cystic fluid were immediately ultrafiltered by means of Amicon Centriflo CF 25 cones. The ultrafiltrates were used for the determination of the free (non-protein bound) platinum concentrations. The original plasma, cystic fluids and ultrafiltrates were stored at −20°C before analysis. Platinum levels were measured by flameless atomic absorption spectrophotometry as described previously (Fish *et al.*, 1984). Plasma and cystic fluid samples containing CDDP were evaluated using pre-infusion fluids spiked with drug, to overcome any matrix interference. The area under the concentration × time curve (AUC) for platinum in plasma and cystic fluid was measured by the trapezoidal method.

The time-course of total platinum in plasma and cystic fluid for the three patients is shown in Figure 1. In both fluids, peak platinum levels occurred at the end of drug infusion, and after 24 h, the total plasma platinum level had fallen to ~80% of the peak values. The maximum total platinum concentration achieved and the calculated AUCs in cystic fluid were variable between patients (Table I). It is known that CDDP or its metabolites bind firmly to plasma proteins (Vermorken *et al.*, 1984). These macromolecular complexes appear to have little demonstrable cytotoxicity *in vitro* (Holdener *et al.*, 1983) and therefore, it would seem that the

Correspondence: R.G. Fish.
Received 31 January 1985; and in revised form, 29 March 1985.

© The Macmillan Press Ltd., 1985

**Figure 1** Concentration of total platinum in plasma (●) and cystic fluid (○) in patients A, B and C receiving a 6 h i.v. infusion of CDDP (—).

**Figure 2** Mean concentrations (±s.e.) of non-protein bound (ultrafilterable) platinum in plasma (●) and cystic fluid (○) in 3 patients receiving CDDP (—).

**Table I** Platinum concentration in cystic fluid and plasma from three patients

| | *Patients* | | |
|---|---|---|---|
| *Parameter* | *A* | *B* | *C* |
| *Dose* ($mg\,m^{-2}$) | 100 | 60 | 60 |
| *Peak concentration* ($\mu g\,ml^{-1}$) | | | |
| Plasma, total platinum | 3.01 | 2.50 | 2.28 |
| Cystic, total platinum | 1.72 | 1.50 | 0.41 |
| Plasma, ultrafilterable platinum | 0.41 | 0.38 | 0.48 |
| Cystic, ultrafilterable platinum | 0.38 | 0.44 | 0.35 |
| $AUC_{0-24}(\mu g\,ml^{-1}h)$ | | | |
| Plasma, total platinum | 50.07 | 43.40 | 35.60 |
| Cystic, total platinum | 25.77 | 21.60 | 5.80 |
| Plasma, ultrafilterable platinum | 2.07 | 2.20 | 3.10 |
| Cystic, ultrafilterable platinum | 1.15 | 1.50 | 1.60 |

direct use of pharmacokinetic data based upon the total achievable plasma platinum levels in patients is inappropriate as a reflection of tumour exposure to active platinum species.

The mean concentration changes for the non-protein bound platinum species in the 3 patients are shown in Figure 2. Peak platinum levels were similar in both fluids and between patients. The AUCs for ultrafilterable platinum in the cystic fluids were ~50% of those for plasma (Table I), although more frequent sampling of this fluid may have provided a better estimate. In both fluids, the non-protein bound platinum declined rapidly during the post-infusion period and was below detection after 3 h.

In these patients, platinum species rapidly diffused into cystic fluid and, with the present dosing schedule, peak levels ranging from 0.38–0.48 $\mu g\,ml^{-1}$ and a tumour exposure (AUC) of 1.15–1.60 $\mu g\,ml^{-1}\,h^{-1}$ were obtained for the pharmacologically active non-protein bound platinum in this fluid. It is concluded that the free platinum concentration is similar in the cystic fluid of all 3 patients, as is that in the plasma. The data support the idea of using only 10% of the peak plasma concentration of total platinum as a reasonable *in vitro* assay concentration (Alberts *et al.*, 1981).

### References

ADAMS, M., KERBY, I.J., ROCKER, I. & JOHANSEN, K. (1984). Combined modality treatment for advanced ovarian cancer. *Clin. Oncol.*, **10**, 80.

ALBERTS, D.S., SALMON, S.E., CHEN, G., MOON, T.E., YOUNG, L. & SURWIT, E.A. (1981). Pharmacologic studies of anticancer drugs with the human tumour stem cell assay. *Cancer Chemother. Pharmacol.*, **6**, 253.

FISH, R.G., SHELLEY, M.D. & GRIFFITHS, H. (1984). Total body clearance and platinum accumulation in patients treated with cis-dichlorodiammine-platinum. *Ther. Drug Monit.*, **6**, 251.

GERSHENSON, D.M., WHARTON, J.T., HERSON, J., EDWARD, C.L. & RUTLEDGE, F.N. (1981). Single-agent cis-patinum therapy for advanced ovarian cancer. *Obstet. Gynecol.*, **58**, 487.

HOLDENER, E.E., PARK, C.H., BELT, R.J., STEPHENS, R.L. & HOOGSTRATEN, B. (1983). Effect of mannitol and plasma on the cytotoxicity of cisplatin. *Eur. J. Cancer Clin. Oncol.*, **19**, 515.

MORASCA, L., ERBA, E., VAGHI, M. & 4 others. (1983). Clinical correlates of *in vitro* drug sensitivities of ovarian cancer cells. *Br. J. Cancer*, **48**, 61.

SALMON, S.E. & VON HOFF, D.D. (1982). *In vitro* evaluation of anticancer drugs with the human tumour stem cell assay. *Recent. Adv. Clin. Oncol.* p. 3.

VERMORKEN, J.B., VAN DER VIJGH, W.J.F., KLEIN, I., HART, A.A.M., GALL, H.E. & PINEDO, H.M. (1984). Pharmacokinetics of free and total platinum species after short-term infusion of cisplatin. *Cancer Treat. Rep.*, **68**, 505.

VON HOFF, D.D. & WEISENTHAL, L. (1980). *In vitro* methods to predict for patient response to chemotherapy. *Adv. Pharmacol Chemother.*, **17**, 133.

VON HOFF, D.D. (1983). Send this patient's tumour for culture and sensitivity. *N. Engl. J. Med.*, **308**, 154.

WILSON, A.P. & NEAL, F.E. (1981). *In vitro* sensitivity of human ovarian tumours to chemotherapeutic agents. *Br. J. Cancer*, **44**, 189.